# Programmed cell death in *Leishmania*: biochemical evidence and role in parasite infectivity

**DOI:** 10.3389/fcimb.2012.00095

**Published:** 2012-07-10

**Authors:** Sreenivas Gannavaram, Alain Debrabant

**Affiliations:** Laboratory of Emerging Pathogens, Division of Emerging and Transfusion Transmitted Diseases, Center for Biologics Evaluation and Research, Food and Drug AdministrationBethesda, MD, USA

**Keywords:** apoptosis, endonuclease, *Leishmania*, metacaspase, programmed cell death, protozoa, trypanosomatid

## Abstract

Demonstration of features of a programmed cell death (PCD) pathway in protozoan parasites initiated a great deal of interest and debate in the field of molecular parasitology. Several of the markers typical of mammalian apoptosis have been shown in *Leishmania* which suggested the existence of an apoptosis like death in these organisms. However, studies to elucidate the downstream events associated with phosphotidyl serine exposure, loss of mitochondrial membrane potential, cytochrome c release, and caspase-like activities in cells undergoing such cell death remain an ongoing challenge. Recent advances in genome sequencing, chemical biology should help to solve some of these challenges. *Leishmania* genetic mutants that lack putative regulators/effectors of PCD pathway should not only help to demonstrate the mechanisms of PCD but also provide tools to better understand the putative role for this pathway in population control and in the establishment of a successful infection of the host.

## Introduction

Programmed cell death (PCD), commonly manifested as apoptosis, plays crucial roles in a multitude of physiological processes starting from embryogenesis to maintenance of the immune system. Evolutionarily, apoptosis emerged along with multicellular organisms, primarily as a host defense mechanism against viral infections (Ameisen, 1996). However, since the first description of a PCD-like pathway in trypanosomatid parasites (Ameisen et al., 1995), increasing experimental evidence has accumulated suggesting that similar processes also appear to exist in many single-celled parasitic organisms including *Plasmodium* species (Al-Olayan et al., 2002; Ch'ng et al., 2010), *Toxoplasma gondii* (Peng et al., 2003), *Trichomonas vaginalis* (Chose et al., 2002), *Entamoeba histolytica* (Villalba et al., 2007), and *Giardia lamblia* (Ghosh et al., 2009). In trypanosomatids, features suggesting PCD have been reported in response to a wide range of stimuli such as heat shock, reactive oxygen species, antiparasitic drugs, prostaglandins, and antimicrobial peptides (Lee et al., 2002; Duszenko et al., 2006; Jimenez-Ruiz et al., 2010). Many biochemical events that accompany mammalian apoptosis such as generation of reactive oxygen species, increase in cytosolic Ca^2+^ levels, alterations in mitochondrial outer membrane potential, exposure of phosphatidylserine (PS) in the outer leaflet of the plasma membrane, release of cytochrome c, activation of caspase-like activities and nucleases that cleave genomic DNA have also been widely documented in trypanosomatid parasites (Sereno et al., 2001; Arnoult et al., 2002; Lee et al., 2002; Mukherjee et al., 2002; Zangger et al., 2002; Debrabant et al., 2003; van Zandbergen et al., 2010). Although autophagy-related processes typically used by cells as a survival mechanism in response to stress have also been shown to lead to cell death under certain conditions (Debnath et al., 2005), their contribution to PCD in parasitic protozoan remains to be elucidated. Therefore, this review will only focus on the evidence for a PCD pathway in *Leishmania* and review the putative molecules involved in such pathway, if adequately demonstrated. We discuss the putative role of PCD in *Leishmania* infectivity and suggest future approaches to better understand the role of such cell death pathway in *Leishmania* and related trypanosomatid parasites.

## Biochemical evidence of programmed cell death in *Leishmania*

### Morphological changes

In metazoan organisms, cell morphology during execution phase of apoptosis is accompanied by characteristic changes that are distinct from other forms of cell death namely autophagy and necrosis (Klionsky et al., 2008; Kroemer et al., 2009; Galluzzi et al., 2012). Loss of cell volume with an intact plasma membrane is considered a hallmark of a cell undergoing apoptotic death unlike necrotic death where such loss of volume is usually a result of non-intact plasma membrane (Kroemer et al., 2009; Galluzzi et al., 2012). *Leishmania* parasites go through a series of distinct morphological shapes and sizes during their life cycle in the insect vector and mammalian hosts. These distinct developmental stages during the normal differentiation of the parasite have been well-characterized (Handman, 1999; Gossage et al., 2003). During its differentiation from procyclic to metacyclic promastigotes in the sand fly vector, the body of the parasite undergoes dramatic shrinkage which is associated with autophagic processes (Besteiro et al., 2006) that do not culminate in cell death. However, the morphological changes observed during *Leishmania* PCD (e.g., cell shrinkage, nuclear condensation) are not well understood; therefore, unlike for metazoans, cell shrinkage cannot be used as a reliable marker of PCD in these organisms.

### Phosphatidylserine exposure at the cell surface

Phospholipid composition in the plasma membrane of mammalian cells is not identical between the two leaflets of the membrane bilayer. The outer leaflet is predominantly composed of choline-containing phospholipids, phosphatidylcholine, and sphingomyelin, whereas the aminophospholipids, phosphatidylethanolamine, and PS populate the inner leaflet (Bevers and Williamson, 2010). This asymmetry in the lipid composition is maintained in quiescent cells by an ATP-dependent mechanism (Tang et al., 1996). However, in apoptotic cells such asymmetry is lost and as a result PS is exposed at the cell surface that can be detected by its reactivity with annexin-V (Martin et al., 1995). This PS exposure was identified as an early event in cells undergoing apoptosis regardless of the stimuli in mammalian apoptosis. Several studies in *Leishmania* reported PS exposure in stationary phase promastigotes and also in response to heat shock, serum deprivation, and a range of chemical inducers based on annexin-V binding to these parasites which is widely used maker of PCD in these organisms (de Freitas Balanco et al., 2001; Jimenez-Ruiz et al., 2010). Moreover, PS-dependent recognition and engulfment of *Leishmania* parasites by mammalian phagocytic host cells have been proposed as a mechanism for invading macrophages and in inducing an anti-inflammatory response by macrophages and dendritic cells (Wanderley et al., 2006). Recently, exposure of PS on *L. amazonensis* parasites derived from skin lesions has been shown to correlate with diffuse cutaneous leishmaniasis compared to localized lesions (França-Costa et al., 2012). Similar PS exposure–dependent mechanism is also utilized by vaccinia virus to enter host cells (Mercer and Helenius, 2008). In mammalian cells, membrane bound protein(s) are considered essential for exposure of PS as a result of collapsed asymmetry in lipid distribution and the protein likely responsible for this membrane scrambling activity was identified as phospholipid scramblase1 (Bevers and Williamson, 2010). However, *Leishmania* and other trypanosomatid parasite genomes do not have an easily identifiable sequence homolog of this protein, underlining the importance of further studies to assess the mechanism of PS exposure to the cell surface of *Leishmania*. In addition, annexin-V is also known to bind anionic phospholipids other than PS. Reagents that could specifically react with PS such as PS-specific monoclonal antibodies or molecular pattern recognition reagents such as aptamers may be of value in confirming that PS is indeed expressed on the cell surface of *Leishmania*.

### Cytochrome c release

In mammalian cells, two major signaling cascades lead to apoptosis. The extrinsic cascade is mediated by activation of tumor necrosis family receptors, also known as death receptors. In contrast, the intrinsic cell death pathway is triggered by stimuli such as cytokine deprivation, DNA damage, and cytotoxic stress and is orchestrated by mitochondria (Brenner and Mak, 2009). The pro-apoptotic Bcl-2 members Bax, Bak, Bid, and others initiate the mitochondrial cell death pathway by permeabilizing the mitochondrial outer membrane. This allows the release of proapoptotic factors from the mitochondria such as cytochrome c, which binds to the adaptor protein Apaf-1 resulting in the activation of caspase-9 (Brenner and Mak, 2009; Pradelli et al., 2010; Abdelwahid et al., 2011; Huttemann et al., 2011). In addition, mitochondrial alterations including disruption of electron transport, oxidative phosphorylation and ATP production, release of other proteins such as Htr2/Omi, Smac/Diablo that trigger caspase activation and changes of cellular redox potential also contribute to the intrinsic PCD pathway (Green and Reed, 1998; Pradelli et al., 2010). Release of cytochrome c has been well documented in many species of *Leishmania* in response to several apoptotic stimuli (Gannavaram et al., 2008; Jimenez-Ruiz et al., 2010). This was also observed when the proapoptotic mammalian Bax protein was exogenously expressed in *Trypanosoma brucei* (Esseiva et al., 2004). However, release of cytochrome c in isolation may be of limited utility as a definite marker of *Leishmania* PCD unless downstream events are better understood. This is because even though cytochrome c is released from the inter membrane space of mitochondria into the cytoplasmic compartment of *Leishmania*, no evidence for downstream events such as binding to Apaf- 1 and activation of caspase-9 homologs has been demonstrated. Homology searches in the *Leishmania* genome databases indicate the presence of a possible homologue of Apaf-1 protein (Table 1). It would be interesting to investigate if this protein indeed has Apaf-1 activity (i.e., bind to *Leishmania* cytochrome c and promote cell death). Mouse genetic studies have shown that Lys72 of cytochrome c is essential for its interaction with Apaf-1, and a mutant lacking the Lys72 can still function in electron transport chain (Hao et al., 2005). However, in a knock-in mouse with this mutation even though Apaf-1 oligomerization did not occur, caspase activation indeed took place suggesting that Apaf-1 may only speed up apoptosis but is not absolutely required for apoptosis to proceed further (Ekert et al., 2004). On the other hand, in yeast, cytochrome c release from mitochondria has been suggested to promote cell death although causal explanation is lacking (Ludovico et al., 2002). These reports suggest that similar to yeast, the release of cytochrome c in *Leishmania* needs further investigation in order to identify the signaling events associated with this molecule that are involved in PCD. This would help to define further a putative intrinsic PCD pathway in *Leishmania.*

**Table 1:** **We searched the literature and conducted BLASTP homology searches, followed by reciprocal best-hit analysis, to assemble a list of *Leishmania* homologs of the putative regulators or effectors of PCD**.

**Human homolog (UniProt)**	**BLASTP reciprocal best-hit**	**High-score**	**Expectation value**	**Reference(s)**
Endonuclease G	LmjF.10.0610	150	4.1e-22	Gannavaram et al., 2008
(Q14249)	LinJ.10.0660	147	3.0e-21	BoseDasgupta et al., 2008
				Rico et al., 2009
TatD deoxyribonuclease	LmjF.11.1280	599	2.2e-59	Gannavaram and Debrabant, 2012
(Q6P1N9)	LinJ.11.1270	593	9.6e-59	
				BoseDasgupta et al., 2008
Flap endonuclease 1	LmjF.27.0250	909	1.6e-92	BoseDasgupta et al., 2008
(FEN1) (P39748)	LinJ.27.0260	905	4.2e-92	
Apoptosis-inducing factor 1	LmjF.32.3310	111	0.00049	none
(AIF1) (O95831)	LinJ.32.3510	114	0.00023	
Apoptotic protease-activating factor 1	LmjF.10.0780	299	2.2e-25	none
(O14727)	LinJ.10.0830	303	5.1e-26	

We identified homologs for five of such regulators or effectors of PCD. Some of these molecules have been characterized in terms of their biochemical activities and role in PCD but others need future studies. We did not identify homologs for any of the other mitochondrial or other organelles based molecules with roles in metazoan apoptosis (http://stke.sciencemag.org/cgi/cm/stkecm; CMP_18019; Bergmann and Steller, 2010).

### Caspase-like and metacaspase activities

Caspases and the members of the Bcl-2 family are the most important regulators of the apoptotic process in metazoans. In protozoan parasites, however, there is very little information about the existence of homologs of the Bcl-2 proteins, even though some indirect evidence indicates that Bcl2-responsive proteins may exist in *Leishmania* (Alzate et al., 2006). On the other hand, extensive evidence for the existence of caspase-like activities associated with parasite PCD has been published (Lee et al., 2002; Paris et al., 2004; Sen et al., 2004; Singh et al., 2005). These groups have reported the activation of proteases able to degrade classical substrates of mammalian caspases in *Leishmania* undergoing PCD. Even though caspase-like activities have been repeatedly reported these protease activities do not appear to be due to bona fide caspases, due to the fact that “caspase” genes have not been found in any of the available complete *Leishmania* genomes and none of the genes encoding the caspase-like enzymatic activities reported in *Leishmania* have been cloned.

Orthologs of caspases, i.e., metacaspases (MCs) and paracaspases, have been identified first from slime mold and later from several plants (Uren et al., 2000). MCs are cysteine proteases with structural similarity to caspases containing a catalytic cysteine histidine dyad. However, biochemical analyses revealed that MCs have distinct substrate specificity (Lys/Arg) from caspases (Asp). A role for MCs in PCD has been reported in plants, yeasts, and protozoan parasites including *Leishmania* (Silva et al., 2005; Lee et al., 2007; Meslin et al., 2007; Coll et al., 2010). Most *Leishmania* species contain a single MC gene except in *L. infantum* and *L. donovani* subtypes where two MCgenes have been found (Lee et al., 2007). Structurally, *Leishmania* MCs contain a central active domain containing the conserved catalytic cysteine and histidine dyad and a proline-rich C-terminal domain (Lee et al., 2007). In *L. donovani*, LdMC1 and LdMC2 cleave arginine/lysine containing substrates without any requirement for proteolytic activation, neither in normal conditions nor upon oxidative stress-induced PCD. LdMCs were reported to be stored in acidocalcisomes and released from these vesicles when cells were treated with hydrogen peroxide (Lee et al., 2007). Further, *Leishmania* overexpressing LdMC1 were more sensitive to hydrogen peroxide treatment suggesting a role in PCD (Lee et al., 2007). In contrast to LdMCs, the *L. major* MC (LmjMC) was reported to be activated by autoprocessing and its recombinant putative catalytic domain was ~300 times more active than the full-length recombinant enzyme for cleaving a fluorogenic substrate containing GGR residues *in vitro* (Gonzalez et al., 2007). LmjMC overexpression was found to enhance *L. major* sensitivity to oxidative stress as measured by the increase of phosphotidyl serine exposure at the parasites surface and the rapid loss of mitochondrial membrane potential, also suggesting a role in *Leishmania* PCD (Gonzalez et al., 2007; Zalila et al., 2011). However, the physiological substrates of *Leishmania* MCs remain to be identified. Interestingly, similar to *Leishmania, Plasmodium* MC has also been shown to exhibit a calcium-dependent, arginine-specific protease activity. Further expression of *P. falciparum* metacsapse in the yeast MC null mutant increased its susceptibility to undergo cell death under oxidative stress (Meslin et al., 2011). A promising approach that could yield valuable information regarding proteins that are substrates for MCs is the activity based probes that have been successfully employed in characterizing several cysteine proteases (Bogyo et al., 2000), kinases (Cohen et al., 2005), and phosphatases (Kumar et al., 2004). Identification of cellular substrates is the first step in further elucidating the role of MC in *Leishmania* PCD. A non-apoptotic role has also been ascribed to LmjMC. LmjMC was found to play a role during organelle segregation and cell-cycle progression under normal physiological conditions (Ambit et al., 2008). In addition, LmjMC null mutants were only viable when LmjMC was re-expressed from an episome at physiological levels, thus reinforcing its importance in parasite survival (Ambit et al., 2008). These observations suggest that *Leishmania* MCs could have separate roles depending on the environmental conditions. Additional studies will be needed to better understand the various roles of MCs in *Leishmania*.

### Nuclease activities

Among the putative effector molecules of *Leishmania* PCD, nucleases have been best characterized. Since no bona fide caspase has been detected in trypanosomatid parasite, caspase-dependent DNase is unlikely to be encoded by their genomes as evidenced by the absence of any homolog of caspase-activated DNase (CAD) in their genomes. In mammalian apoptosis, two endonucleases are known to be involved in DNA fragmentations that do not require activation by caspases. These are apoptosis inducing factor (AIF) and endonuclease G (EndoG). EndoG has been shown to be a mitochondrial enzyme that is released in response to inducers of apoptosis and is involved in DNA degradation of dying cells (Li et al., 2001). In *Leishmania*, EndoG-mediated DNA degradation has been independently demonstrated in three different laboratories using hydrogen peroxide or pharmacological agents (BoseDasgupta et al., 2008; Gannavaram et al., 2008; Rico et al., 2009). The overexpression of EndoG in *Leishmania* resulted in spontaneous DNA fragmentation in amastigotes and not in the promastigotes, suggesting that additional factors necessary for efficient DNA degradation are expressed in a stage-specific manner (Gannavaram et al., 2008). In *L. major*, oxidative stress associated with the differentiation process induced high levels of PCD when EndoG is overexpressed in *L. major* parasites (Gannavaram et al., 2008). Also, induction of oxidative burst in macrophages by LPS and IFN-γ treatment triggered PCD in intracellular amastigotes compared to non-stimulated host cells indicating that oxidative burst can induce PCD even in wild type parasites. These results suggested a role for EndoG in *Leishmania* PCD.

Functional genomic studies in *C. elegans* showed that several nucleases are involved in DNA degradation observed during apoptosis (Parrish and Xue, 2003). They include nuc-1, cps-6, AIF, cell death-related nucleases 1–6, and cyclophilin-13 (Parrish et al., 2001; Wang et al., 2002; Parrish and Xue, 2003). In *Leishmania*, indirect evidence is presented for a role of additional nucleases such as TatD-related nuclease and flap endonuclease in DNA degradation during PCD (BoseDasgupta et al., 2008). Recently, we described the involvement of TatD nuclease during PCD in the protozoan parasite *Trypanosoma brucei*. *T. brucei* TatD nuclease showed intrinsic DNase activity was localized in the cytoplasm and translocated to the nucleus where it could interact with EndoG when cells were treated with inducers that cause PCD. Over-expression of TatD nuclease resulted in elevated PCD and conversely, loss of TatD expression by RNAi conferred significant resistance to the induction of PCD in *T. brucei* (Gannavaram and Debrabant, 2012). These results show that *T. brucei* overexpressing TatD spontaneously undergo PCD under conditions that the parasites are likely to encounter in a human host, supporting a role of TatD in PCD in trypanosomatid parasites.

An AIF homolog has been shown to be translocated from the mitochondria to the nucleus after the onset of PCD in *Dictyostelium discoideum* (Arnoult et al., 2001). Recent searches in the *Leishmania* genomes suggested the presence of a weak homolog of AIF (Table 1). It would be of interest to investigate if this molecule has nuclease activity associated with PCD. Additionally, a recent report demonstrated in *C. elegans* the role of DICER in mediating DNA strand breaks during cell death associated with the normal development of this organism (Nakagawa et al., 2010). DICER usually degrades double stranded RNA into small RNAs that are involved in gene silencing. The unusual change in substrate specificity of DICER from a dsRNA to a DNA is accompanied by proteolytic cleavage of its carboxy terminal region (Nakagawa et al., 2010). In comparison to other eukaryotic cells, most *Leishmania* species are known to have lost the machinery required for dsRNA-mediated RNA interference except *L. braziliensis* and other species within the *Leishmania* subgenus *Viannia* (Lye et al., 2010). It would be of interest to investigate if *L. braziliensis* DICER has DNase activity that is pertinent to PCD. In addition, since DICER activity has also been demonstrated in *T. brucei* (Shi et al., 2006), the putative role of this molecule in *T. brucei* PCD could be an interesting avenue to pursue.

## Role of *Leishmania* PCD in parasite infectivity and survival

### Population control

Several arguments have been made recently about the existence of a PCD pathway and the physiological functions that such pathway might serve in the life cycle of unicellular parasites. For instance, PCD is considered useful in regulating the parasite cell density in the host as a mean of avoiding hyperparasitism. In *Plasmodium*, a PCD-like mechanism was suggested as a modality for limiting parasite density in the mosquito and in the mammalian hosts (Al-Olayan et al., 2002; Deponte and Becker, 2004). *T. brucei* parasites have been hypothesized to utilize PCD to regulate their cell densities in the insect vector and in mammalian host in response to the changing host antibody repertoire because of the variations in antigenic composition on its surface (Welburn and Maudlin, 1997; Vassella et al., 1997). Further, it has been shown that addition of prostaglandin D2 to cultures inhibited growth of bloodstream form parasites and induced an apoptosis-like cell death (Figarella et al., 2005). These observations led to the hypothesis that the high serum prostaglandin levels in the mammalian host may play a role in regulating parasite densities by inducing cell death (Figarella et al., 2005; Duszenko et al., 2006). However, definitive evidence that links the role of PCD in controlling parasite density in either mammalian or invertebrate hosts for *Plasmodium* or *T. brucei* is lacking. Similar evidence demonstrating a role for PCD in the regulation of parasite population is also lacking in *Leishmania* even though apoptotic features have been widely demonstrated in these parasites (Luder et al., 2010). To that end, the development of genetic mutants lacking the putative regulators/effectors of *Leishmania* PCD described so far (e.g., MC or EndoG null mutants) would be extremely useful to evaluate the role of parasite PCD in maintaining host–parasite equilibrium.

### Modulation of host immunity

PCD has also been considered to influence the outcome of an infection during the early phase of interactions between parasites and their mammalian host. Recently it has been proposed that for establishing a successful *L. major* infection in mice, the presence of apoptotic parasites in the inoculum was a key determinant (van Zandbergen et al., 2006, 2010). This was based on the observation that presence of annexin-V binding parasites within the inoculum leads to increased uptake by neutrophils that are attracted to the site of infection before the macrophages home in van Zandbergen et al. (2004, 2006). This was accompanied by the release of anti-inflammatory cytokines such as TGF-β, IL-10 and lipids such as lipoxinA4 and down-regulation of pro-inflammatory cytokines such as TNF-α and lipids like leukotriene-B4, which could favor parasite survival. In addition, in the *L. major* infection, when the annexin-V binding parasites were depleted from the inoculum, the presumably non-apoptotic parasites had limited virulence as indicated by the reduced size of the lesions. Further, the release of the anti-inflammatory cytokine TGF-β by the neutrophils correlated with the dose of the annexin-V binding parasites in the inoculum. Also there was an inverse correlation between the annexin-V binding parasites and the secretion of pro-inflammatory cytokine TNF-α. (van Zandbergen et al., 2006). These observations led to the hypothesis that the “eat-me” signal represented by the annexin-V binding on the parasites surface promotes immunologically “silent” uptake of the *Leishmania* parasites by macrophages and dendritic cells, as was observed in other immunosuppressive effects of apoptotic cells in mammalian homeostatic cell death (Huynh et al., 2002; van Zandbergen et al., 2006; Obeid et al., 2007). For this silent uptake to happen, the parasites in the inoculum must be able to recruit phagocytic cells to the inoculation site since the phagocytes require chemotactic guidance. There is some evidence to indicate that neutrophils are recruited by a *Leishmania* chemotactic factor and this activity is likely present in the lipid fraction of the parasites (van Zandbergen et al., 2007). Additional chemotactic signals such as lysophosphatidylcholine, sphingosine 1-phosphate and CX3CL1/fractalkine have been described for their potential to recruit professional phagocytes such as macrophages (Li, 2012). However, roles of such additional “find me” signals in the context of a *Leishmania* infection remain to be investigated. In a *Leishmania* infection, neutrophils migrate to the site within 40 min and localize around bite sites (Peters et al., 2008). The neutrophils recognize the phosphotidyl serine exposed on a subpopulation in the metacyclics and phagocytose the cells in the inoculum in a non-immunogenic mechanism.

Recently, calreticulin, mostly know as an endoplasmic reticulum (ER) chaperone protein, was shown to also function as “eat me” signals (Obeid et al., 2007). Calreticulin was shown to be upregulated on the surface of apoptotic cancer cells that favored their uptake by phagocytic cells in a non-immunogenic “silent” mechanism (Obeid et al., 2007; Martins et al., 2010). The role of calreticulin as an ER chaperone molecule has been previously characterized in *Leishmania* (Debrabant et al., 2002). Whether this protein is also exposed at the surface of *Leishmania* to facilitate their silent entry into the host phagocytic cells needs to be investigated. Additionally, mannose binding lectins or lung surfactant proteins A and D have also been shown to act as eat-me signals (Ogden et al., 2001; Vandivier et al., 2002). In contrast, negative regulators of phagocytosis such as lactoferrin have also been described (Bournazou et al., 2009). All this diversity of signals indicates that phagocytosis is a finely regulated process with broad physiological effects and in principle parasites might utilize many signals to gain silent entry into a host cell. Therefore, it would be pertinent to explore such signaling pathways that promote non-immunogenic uptake of *Leishmania*.

## Future perspectives

Analysis of genetic mutants facilitated robust demonstration of the existence of genetically PCD pathways in multicellular organisms and retention of those pathways in multicellular organisms is clearly understood from an evolutionary stand point. However, the selection of a PCD pathway in unicellular organisms such as *Leishmania* is less clear and remains to be explained from an evolutionary point of view. Studies in yeast suggested arguments in support of a PCD pathway in single cell organisms. For instance, in co-culture experiments, yeast persistently infected with dsRNA viruses were shown to induce PCD in uninfected yeast cells (Ivanovska and Hardwick, 2005; Schmitt and Reiter, 2008). PCD did not occur in a yeast mutant lacking MC, demonstrating the existence of this conserved cell death pathway in a single cell organism. Recently, *Leishmania* parasites infected with a dsRNA virus have been shown to cause metastatic spreading of the otherwise localized cutaneous lesions (Ives et al., 2011). This metastasis involved modulation of host immunity by viral RNA that resulted in heightened host pro-inflammatory responses (Ronet et al., 2011). Studies in virally infected *Leishmania* parasites analogous to those in yeast might help clarify the existence of a PCD pathway in *Leishmania* parasites and support its selection in single cell organisms.

In comparison to *C. elegans* and yeast, studies elucidating molecular mechanisms of PCD in trypanosomatid parasites are limited primarily because of the apparent absence of homologues to key regulatory or effector molecules of apoptosis in the trypanosomatid genomes that have been described in mammalian or nematode apoptosis such as Bcl-2 family members and caspases (Smirlis et al., 2010). Searches in annotated *Leishmania* genomes reveal that homology based searches offer limited clues in attempts to deciphering the mechanisms mediating PCD machinery in these parasites (Table 1). The absence of homologues of key regulatory or effector molecules of mammalian apoptosis in trypanosomatid parasites suggests that their PCD pathways are probably less evolved than apoptotic pathways of mammalian cells. Although still poorly understood, the existence of conserved PCD pathways in trypanosomatid parasites can provide targets for developing novel chemotherapies. Recent pharmacological studies elicited interest in several molecules with activities that trigger apoptotic death in cancerous cells as potential antiparasitic agents (Fuertes et al., 2008).

Studies with genetic mutants that lack regulators/effectors of PCD (e.g., MC or EndoG null mutants) would clarify the role of each of these proteins in *Leishmania* PCD. However, this approach is contingent upon the fact that analysis of such mutants can yield evidence for a role in PCD under appropriate settings, as the same molecule may have multiple functions in the cell including functions unrelated to PCD. For instance, EndoG null mutant mice have been shown to be associated with impaired mitochondrial respiration and increased production of reactive oxygen species, indicating that pro-apoptotic activities of this nuclease are likely to be specialized functions for this protein (David et al., 2006; McDermott-Roe et al., 2011). Similarly, studies using *Leishmania* genetic mutants lacking regulators/effectors of PCD may help better understand the role of this pathway in the survival of the parasite population in its invertebrate host. For example, to determine if PCD of non-metacyclic *Leishmania* parasites occurs in an infected sand fly and contributes to the survival of infectious metacyclic forms which is the only stage that is transmitted and can establish a successful infection in the mammalian host and therefore ensure the propagation of the species.

In summary, our knowledge of *Leishmania* PCD is still very fragmented. In the last ten years, several putative effector molecules of such pathway have been identified and characterized to various degrees. Similar effector molecules have also been shown to be involved in PCD pathways in other protozoan parasites suggesting that a common pathway might be conserved among this group of organisms. So far molecules regulating this pathway are unknown. In *Leishmania* the mitochondria appears to play a central role in this pathway (e.g., release of cytochrome c and proapoptotic nucleases) and therefore displays similarities to the intrinsic apoptosis pathway of mammalian cells. Although experimental observations are limited, there is increasing evidence to support the idea that protozoan parasites use PCD for controlling their population in the infected host. Possibly, *Leishmania* could use PCD either in the insect vector to favor the survival of infectious metacyclic forms, or in the mammalian host to avoid hyperparasitism that would prematurely kill the host. In addition, recent evidences suggest that *Leishmania* also exploits features of PCD to facilitate its silent entry in the mammalian host and establish a successful infection. However, there is much work ahead to decipher the multiple roles played by PCD in the biology of *Leishmania*.

### Conflict of interest statement

The authors declare that the research was conducted in the absence of any commercial or financial relationships that could be construed as a potential conflict of interest.

## References

[B1] Abdelwahid E. Rolland S. Teng X. Conradt B. Hardwick J. M. White K. (2011). Mitochondrial involvement in cell death of non-mammalian eukaryotes. Biochim. Biophys. Acta 1813, 597–607 10.1016/j.bbamcr.2010.10.008 20950655 PMC3033473

[B2] Al-Olayan E. M. Williams G. T. Hurd H. (2002). Apoptosis in the malaria protozoan, *Plasmodium berghei*: a possible mechanism for limiting intensity of infection in the mosquito. Int. J. Parasitol. 32, 1133–1143 10.1016/S0020-7519(02)00087-5 12117496

[B3] Alzate J. F. Alvarez-Barrientos A. Gonzalez V. M. Jimenez-Ruiz A. (2006). Heat-induced programmed cell death in *Leishmania infantum* is reverted by Bcl-X(L) expression. Apoptosis 11, 161–171 10.1007/s10495-006-4570-z 16502255

[B4] Ambit A. Fasel N. Coombs G. H. Mottram J. C. (2008). An essential role for the *Leishmania major* metacaspase in cell cycle progression. Cell Death Differ. 15, 113–122 10.1038/sj.cdd.4402232 17901875

[B5] Ameisen J. C. (1996). The origin of programmed cell death. Science 272, 1278–1279 10.1126/science.272.5266.1278 8650538

[B6] Ameisen J. C. Idziorek T. Billaut-Mulot O. Loyens M. Tissier J. P. Potentier A. Ouaissi A. (1995). Apoptosis in a unicellular eukaryote (*Trypanosoma cruzi*): implications for the evolutionary origin and role of programmed cell death in the control of cell proliferation, differentiation and survival. Cell Death Differ. 2, 285–300 17180034

[B7] Arnoult D. Akarid K. Grodet A. Petit P. X. Estaquier J. Ameisen J. C. (2002). On the evolution of programmed cell death: apoptosis of the unicellular eukaryote *Leishmania major* involves cysteine proteinase activation and mitochondrion permeabilization. Cell Death Differ. 9, 65–81 10.1038/sj.cdd.4400951 11803375

[B8] Arnoult D. Tatischeff I. Estaquier J. Girard M. Sureau F. Tissier J. P. Grodet A. Dellinger M. Traincard F. Kahn A. Ameisen J. C. Petit P. X. (2001). On the evolutionary conservation of the cell death pathway: mitochondrial release of an apoptosis-inducing factor during *Dictyostelium discoideum* cell death. Mol. Biol. Cell 12, 3016–3030 11598188 10.1091/mbc.12.10.3016 PMC60152

[B9] Bergmann A. Steller H. (2010). Apoptosis, stem cells, and tissue regeneration. Sci. Signal. 3, re8 10.1126/scisignal.3145re8 20978240 PMC2991142

[B10] Besteiro S. Williams R. A. Morrison L. S. Coombs G. H. Mottram J. C. (2006). Endosome sorting and autophagy are essential for differentiation and virulence of *Leishmania major*. J. Biol. Chem. 281, 11384–11396 10.1074/jbc.M512307200 16497676

[B11] Bevers E. M. Williamson P. L. (2010). Phospholipid scramblase: an update. FEBS Lett. 584, 2724–2730 10.1016/j.febslet.2010.03.020 20302864

[B12] Bogyo M. Verhelst S. Bellingard-Dubouchaud V. Toba S. Greenbaum D. (2000). Selective targeting of lysosomal cysteine proteases with radiolabeled electrophilic substrate analogs. Chem. Biol. 7, 27–38 10.1016/S1074-5521(00)00061-2 10662686

[B13] BoseDasgupta S. Das B. B. Sengupta S. Ganguly A. Roy A. Dey S. Tripathi G. Dinda B. Majumder H. K. (2008). The caspase-independent algorithm of programmed cell death in *Leishmania* induced by baicalein: the role of LdEndoG, LdFEN-1 and LdTatD as a DNA ‘degradesome’. Cell Death Differ. 15, 1629–1640 10.1038/cdd.2008.85 18566607

[B14] Bournazou I. Pound J. D. Duffin R. Bournazos S. Melville L. A. Brown S. B. Rossi A. G. Gregory C. D. (2009). Apoptotic human cells inhibit migration of granulocytes via release of lactoferrin. J. Clin. Invest. 119, 20–32 10.1172/JCI36226 19033648 PMC2613454

[B15] Brenner D. Mak T. W. (2009). Mitochondrial cell death effectors. Curr. Opin. Cell Biol. 21, 871–877 10.1016/j.ceb.2009.09.004 19822411

[B16] Ch'ng J. H. Kotturi S. R. Chong A. G. Lear M. J. Tan K. S. (2010). A programmed cell death pathway in the malaria parasite *Plasmodium falciparum* has general features of mammalian apoptosis but is mediated by clan CA cysteine proteases. Cell Death Dis. 1, e26 10.1038/cddis.2010.2 21364634 PMC3032337

[B17] Chose O. Noel C. Gerbod D. Brenner C. Viscogliosi E. Roseto A. (2002). A form of cell death with some features resembling apoptosis in the amitochondrial unicellular organism *Trichomonas vaginalis*. Exp. Cell Res. 276, 32–39 10.1006/excr.2002.5496 11978006

[B18] Cohen M. S. Zhang C. Shokat K. M. Taunton J. (2005). Structural bioinformatics-based design of selective, irreversible kinase inhibitors. Science 308, 1318–1321 10.1126/science1108367 15919995 PMC3641834

[B19] Coll N. S. Vercammen D. Smidler A. Clover C. Van Breusegem F. Dangl J. L. Epple P. (2010). *Arabidopsis* type I metacaspases control cell death. Science 330, 1393–1397 10.1126/science.1194980 21097903

[B20] David K. K. Sasaki M. Yu S. W. Dawson T. M. Dawson V. L. (2006). EndoG is dispensable in embryogenesis and apoptosis. Cell Death Differ. 13, 1147–1155 10.1038/sj.cdd.4401787 16239930

[B21] Debnath J. Baehrecke E. H. Kroemer G. (2005). Does autophagy contribute to cell death? Autophagy 1, 66–74 16874022 10.4161/auto.1.2.1738

[B22] Debrabant A. Lee N. Bertholet S. Duncan R. Nakhasi H. L. (2003). Programmed cell death in trypanosomatids and other unicellular organisms. Int. J. Parasitol. 33, 257–267 10.1016/S0020-7519(03)00008-0 12670511

[B23] Debrabant A. Lee N. Pogue G. P. Dwyer D. M. Nakhasi H. L. (2002). Expression of calreticulin P-domain results in impairment of secretory pathway in *Leishmania donovani* and reduced parasite survival in macrophages. Int. J. Parasitol. 32, 1423–1434 10.1016/S0020-7519(02)00134-0 12350377

[B24] de Freitas Balanco J. M. Moreira M. E. Bonomo A. Bozza P. T. Amarante-Mendes G. Pirmez C. Barcinski M. A. (2001). Apoptotic mimicry by an obligate intracellular parasite downregulates macrophage microbicidal activity. Curr. Biol. 11, 1870–1873 10.1016/S0960-9822(01)00563-2 11728310

[B25] Deponte M. Becker K. (2004). *Plasmodium falciparum*–do killers commit suicide? Trends Parasitol. 20, 165–169 10.1016/j.pt.2004.01.012 15099555

[B26] Duszenko M. Figarella K. Macleod E. T. Welburn S. C. (2006). Death of a trypanosome: a selfish altruism. Trends Parasitol. 22, 536–542 10.1016/j.pt.2006.08.010 16942915

[B27] Ekert P. G. Read S. H. Silke J. Marsden V. S. Kaufmann H. Hawkins C. J. Gerl R. Kumar S. Vaux D. L. (2004). Apaf-1 and caspase-9 accelerate apoptosis, but do not determine whether factor-deprived or drug-treated cells die. J. Cell Biol. 165, 835–842 10.1083/jcb.200312031 15210730 PMC2172390

[B28] Esseiva A. C. Chanez A. L. Bochud-Allemann N. Martinou J. C. Hemphill A. Schneider A. (2004). Temporal dissection of Bax-induced events leading to fission of the single mitochondrion in *Trypanosoma brucei*. EMBO Rep. 5, 268–273 10.1038/sj.embor.7400095 14968134 PMC1299006

[B29] Figarella K. Rawer M. Uzcategui N. L. Kubata B. K. Lauber K. Madeo F. Wesselborg S. Duszenko M. (2005). Prostaglandin D2 induces programmed cell death in *Trypanosoma brucei* bloodstream form. Cell Death Differ. 12, 335–346 10.1038/sj.cdd.4401564 15678148

[B30] França-Costa J. Wanderley J. L. Deolindo P. Zarattini J. B. Costa J. Soong L. Barcinski M. A. Barral A. Borges V. M. (2012). Exposure of phosphatidylserine on *Leishmania amazonensis* isolates is associated with diffuse cutaneous leishmaniasis and parasite infectivity. PLoS ONE 7:e36595 10.1371/journal.pone.0036595 22574191 PMC3344919

[B31] Fuertes M. A. Nguewa P. A. Castilla J. Alonso C. Perez J. M. (2008). Anticancer compounds as leishmanicidal drugs: challenges in chemotherapy and future perspectives. Curr. Med. Chem. 15, 433–439 18288998 10.2174/092986708783503221

[B32] Galluzzi L. Vitale I. Abrams J. M. Alnemri E. S. Baehrecke E. H. Blagosklonny M. V. Dawson T. M. Dawson V. L. El-Deiry W. S. Fulda S. Gottlieb E. Green D. R. Hengartner M. O. Kepp O. Knight R. A. Kumar S. Lipton S. A. Lu X. Madeo F. Malorni W. Mehlen P. Nunez G. Peter M. E. Piacentini M. Rubinsztein D. C. Shi Y. Simon H. U. Vandenabeele P. White E. Yuan J. Zhivotovsky B. Melino G. Kroemer G. (2012). Molecular definitions of cell death subroutines: recommendations of the Nomenclature Committee on Cell Death 2012. Cell Death Differ. 19, 107–120 10.1038/cdd.2011.96 21760595 PMC3252826

[B33] Gannavaram S. Debrabant A. (2012). Involvement of TatD nuclease during programmed cell death in the protozoan parasite *Trypanosoma brucei*. Mol. Microbiol. 83, 926–935 10.1111/j.1365-2958.2012.07978.x 22288397

[B34] Gannavaram S. Vedvyas C. Debrabant A. (2008). Conservation of the pro-apoptotic nuclease activity of endonuclease G in unicellular trypanosomatid parasites. J. Cell Sci. 121, 99–109 10.1242/jcs.014050 18073240

[B35] Ghosh E. Ghosh A. Ghosh A. N. Nozaki T. Ganguly S. (2009). Oxidative stress-induced cell cycle blockage and a protease-independent programmed cell death in microaerophilic *Giardia lamblia*. Drug Des. Dev. Ther. 3, 103–110 10.2147/DDDT.S5270 19920926 PMC2769235

[B36] Gonzalez I. J. Desponds C. Schaff C. Mottram J. C. Fasel N. (2007). *Leishmania major* metacaspase can replace yeast metacaspase in programmed cell death and has arginine-specific cysteine peptidase activity. Int. J. Parasitol. 37, 161–172 10.1016/j.ijpara.2006.10.004 17107676

[B37] Gossage S. M. Rogers M. E. Bates P. A. (2003). Two separate growth phases during the development of *Leishmania* in sand flies: implications for understanding the life cycle. Int. J. Parasitol. 33, 1027–1034 10.1016/S0020-7519(03)00142-5 13129524 PMC2839921

[B38] Green D. R. Reed J. C. (1998). Mitochondria and apoptosis. Science 281, 1309–1312 10.1126/science.281.5381.1309 9721092

[B39] Handman E. (1999). Cell biology of *Leishmania*. Adv. Parasitol. 44, 1–39 10563394 10.1016/s0065-308x(08)60229-8

[B40] Hao Z. Duncan G. S. Chang C. C. Elia A. Fang M. Wakeham A. Okada H. Calzascia T. Jang Y. You-Ten A. Yeh W. C. Ohashi P. Wang X. Mak T. W. (2005). Specific ablation of the apoptotic functions of cytochrome C reveals a differential requirement for cytochrome C and Apaf-1 in apoptosis. Cell 121, 579–591 10.1016/j.cell.2005.03.016 15907471

[B41] Huttemann M. Pecina P. Rainbolt M. Sanderson T. H. Kagan V. E. Samavati L. Doan J. W. Lee I. (2011). The multiple functions of cytochrome c and their regulation in life and death decisions of the mammalian cell: from respiration to apoptosis. Mitochondrion 11, 369–381 10.1016/j.mito.2011.01.010 21296189 PMC3075374

[B42] Huynh M. L. Fadok V. A. Henson P. M. (2002). Phosphatidylserine-dependent ingestion of apoptotic cells promotes TGF-beta1 secretion and the resolution of inflammation. J. Clin. Invest. 109, 41–50 10.1172/JCI11638 11781349 PMC150814

[B43] Ivanovska I. Hardwick J. M. (2005). Viruses activate a genetically conserved cell death pathway in a unicellular organism. J. Cell Biol. 170, 391–399 10.1083/jcb.200503069 16061692 PMC2171480

[B44] Ives A. Ronet C. Prevel F. Ruzzante G. Fuertes-Marraco S. Schutz F. Zangger H. Revaz-Breton M. Lye L. F. Hickerson S. M. Beverley S. M. Acha-Orbea H. Launois P. Fasel N. Masina S. (2011). *Leishmania* RNA virus controls the severity of mucocutaneous leishmaniasis. Science 331, 775–778 10.1126/science.1199326 21311023 PMC3253482

[B45] Jimenez-Ruiz A. Alzate J. F. Macleod E. T. Luder C. G. Fasel N. Hurd H. (2010). Apoptotic markers in protozoan parasites. Parasit. Vectors 3, 104 10.1186/1756-3305-3-104 21062457 PMC2993696

[B46] Klionsky D. J. Abeliovich H. Agostinis P. Agrawal D. K. Aliev G. Askew D. S. Baba M. Baehrecke E. H. Bahr B. A. Ballabio A. Bamber B. A. Bassham D. C. Bergamini E. Bi X. Biard-Piechaczyk M. Blum J. S. Bredesen D. E. Brodsky J. L. Brumell J. H. Brunk U. T. Bursch W. Camougrand N. Cebollero E. Cecconi F. Chen Y. Chin L. S. Choi A. Chu C. T. Chung J. Clarke P. G. Clark R. S. Clarke S. G. Clave C. Cleveland J. L. Codogno P. Colombo M. I. Coto-Montes A. Cregg J. M. Cuervo A. M. Debnath J. Demarchi F. Dennis P. B. Dennis P. A. Deretic V. Devenish R. J. Di Sano F. Dice J. F. Difiglia M. Dinesh-Kumar S. Distelhorst C. W. Djavaheri-Mergny M. Dorsey F. C. Droge W. Dron M. Dunn W. A. Jr. Duszenko M. Eissa N. T. Elazar Z. Esclatine A. Eskelinen E. L. Fesus L. Finley K. D. Fuentes J. M. Fueyo J. Fujisaki K. Galliot B. Gao F. B. Gewirtz D. A. Gibson S. B. Gohla A. Goldberg A. L. Gonzalez R. Gonzalez-Estevez C. Gorski S. Gottlieb R. A. Haussinger D. He Y. W. Heidenreich K. Hill J. A. Hoyer-Hansen M. Hu X. Huang W. P. Iwasaki A. Jaattela M. Jackson W. T. Jiang X. Jin S. Johansen T. Jung J. U. Kadowaki M. Kang C. Kelekar A. Kessel D. H. Kiel J. A. Kim H. P. Kimchi A. Kinsella T. J. Kiselyov K. Kitamoto K. Knecht E. Komatsu M. Kominami E. Kondo S. Kovacs A. L. Kroemer G. Kuan C. Y. Kumar R. Kundu M. Landry J. Laporte M. Le W. Lei H. Y. Lenardo M. J. Levine B. Lieberman A. Lim K. L. Lin F. C. Liou W. Liu L. F. Lopez-Berestein G. Lopez-Otin C. Lu B. Macleod K. F. Malorni W. Martinet W. Matsuoka K. Mautner J. Meijer A. J. Melendez A. Michels P. Miotto G. Mistiaen W. P. Mizushima N. Mograbi B. Monastyrska I. Moore M. N. Moreira P. I. Moriyasu Y. Motyl T. Munz C. Murphy L. O. Naqvi N. I. Neufeld T. P. Nishino I. Nixon R. A. Noda T. Nurnberg B. Ogawa M. Oleinick N. L. Olsen L. J. Ozpolat B. Paglin S. Palmer G. E. Papassideri I. Parkes M. Perlmutter D. H. Perry G. Piacentini M. Pinkas-Kramarski R. Prescott M. Proikas-Cezanne T. Raben N. Rami A. Reggiori F. Rohrer B. Rubinsztein D. C. Ryan K. M. Sadoshima J. Sakagami H. Sakai Y. Sandri M. Sasakawa C. Sass M. Schneider C. Seglen P. O. Seleverstov O. Settleman J. Shacka J. J. Shapiro I. M. Sibirny A. Silva-Zacarin E. C. Simon H. U. Simone C. Simonsen A. Smith M. A. Spanel-Borowski K. Srinivas V. Steeves M. Stenmark H. Stromhaug P. E. Subauste C. S. Sugimoto S. Sulzer D. Suzuki T. Swanson M. S. Tabas I. Takeshita F. Talbot N. J. Talloczy Z. Tanaka K. Tanida I. Taylor G. S. Taylor J. P. Terman A. Tettamanti G. Thompson C. B. Thumm M. Tolkovsky A. M. Tooze S. A. Truant R. Tumanovska L. V. Uchiyama Y. Ueno T. Uzcategui N. L. van der Klei I. Vaquero E. C. Vellai T. Vogel M. W. Wang H. G. Webster P. Wiley J. W. Xi Z. Xiao G. Yahalom J. Yang J. M. Yap G. Yin X. M. Yoshimori T. Yu L. Yue Z. Yuzaki M. Zabirnyk O. Zheng X. Zhu X. Deter R. L. (2008). Guidelines for the use and interpretation of assays for monitoring autophagy in higher eukaryotes. Autophagy 4, 151–175 18188003 10.4161/auto.5338 PMC2654259

[B47] Kroemer G. Galluzzi L. Vandenabeele P. Abrams J. Alnemri E. S. Baehrecke E. H. Blagosklonny M. V. El-Deiry W. S. Golstein P. Green D. R. Hengartner M. Knight R. A. Kumar S. Lipton S. A. Malorni W. Nunez G. Peter M. E. Tschopp J. Yuan J. Piacentini M. Zhivotovsky B. Melino G. (2009). Classification of cell death: recommendations of the Nomenclature Committee on Cell Death (2009). Cell Death Differ. 16, 3–11 10.1038/cdd.2008.150 18846107 PMC2744427

[B48] Kumar S. Zhou B. Liang F. Wang W. Q. Huang Z. Zhang Z. Y. (2004). Activity-based probes for protein tyrosine phosphatases. Proc. Natl. Acad. Sci. U.S.A. 101, 7943–7948 10.1073/pnas.0402323101 15148367 PMC419536

[B49] Lee N. Bertholet S. Debrabant A. Muller J. Duncan R. Nakhasi H. L. (2002). Programmed cell death in the unicellular protozoan parasite *Leishmania*. Cell Death Differ. 9, 53–64 10.1038/sj.cdd.4400952 11803374

[B50] Lee N. Gannavaram S. Selvapandiyan A. Debrabant A. (2007). Characterization of metacaspases with trypsin-like activity and their putative role in programmed cell death in the protozoan parasite *Leishmania*. Eukaryot. Cell 6, 1745–1757 10.1128/EC.00123-07 17715367 PMC2043384

[B51] Li L. Y. Luo X. Wang X. (2001). Endonuclease G is an apoptotic DNase when released from mitochondria. Nature 412, 95–99 10.1038/35083620 11452314

[B52] Li W. (2012). Eat-me signals: keys to molecular phagocyte biology and “appetite” control. J. Cell Physiol. 227, 1291–1297 10.1002/jcp.22815 21520079 PMC3242927

[B53] Luder C. G. Campos-Salinas J. Gonzalez-Rey E. van Zandbergen G. (2010). Impact of protozoan cell death on parasite-host interactions and pathogenesis. Parasit. Vectors 3, 116 10.1186/1756-3305-3-116 21126352 PMC3003647

[B54] Ludovico P. Rodrigues F. Almeida A. Silva M. T. Barrientos A. Corte-Real M. (2002). Cytochrome c release and mitochondria involvement in programmed cell death induced by acetic acid in *Saccharomyces cerevisiae*. Mol. Biol. Cell 13, 2598–2606 10.1091/mbc.E01-12-0161 12181332 PMC117928

[B55] Lye L. F. Owens K. Shi H. Murta S. M. Vieira A. C. Turco S. J. Tschudi C. Ullu E. Beverley S. M. (2010). Retention and loss of RNA interference pathways in trypanosomatid protozoans. PLoS Pathog. 6:e1001161 10.1371/journal.ppat.1001161 21060810 PMC2965760

[B56] Martin S. J. Reutelingsperger C. P. McGahon A. J. Rader J. A. van Schie R. C. LaFace D. M. Green D. R. (1995). Early redistribution of plasma membrane phosphatidylserine is a general feature of apoptosis regardless of the initiating stimulus: inhibition by overexpression of Bcl-2 and Abl. J. Exp. Med. 182, 1545–1556 7595224 10.1084/jem.182.5.1545 PMC2192182

[B57] Martins I. Kepp O. Galluzzi L. Senovilla L. Schlemmer F. Adjemian S. Menger L. Michaud M. Zitvogel L. Kroemer G. (2010). Surface-exposed calreticulin in the interaction between dying cells and phagocytes. Ann. N.Y. Acad. Sci. 1209, 77–82 10.1111/j.1749-6632.2010.05740.x 20958319

[B58] McDermott-Roe C. Ye J. Ahmed R. Sun X. M. Serafin A. Ware J. Bottolo L. Muckett P. Canas X. Zhang J. Rowe G. C. Buchan R. Lu H. Braithwaite A. Mancini M. Hauton D. Marti R. Garcia-Arumi E. Hubner N. Jacob H. Serikawa T. Zidek V. Papousek F. Kolar F. Cardona M. Ruiz-Meana M. Garcia-Dorado D. Comella J. X. Felkin L. E. Barton P. J. Arany Z. Pravenec M. Petretto E. Sanchis D. Cook S. A. (2011). Endonuclease G is a novel determinant of cardiac hypertrophy and mitochondrial function. Nature 478, 114–118 10.1038/nature10490 21979051 PMC3189541

[B59] Mercer J. Helenius A. (2008). Vaccinia virus uses macropinocytosis and apoptotic mimicry to enter host cells. Science 320, 531–535 10.1126/science.1155164 18436786

[B60] Meslin B. Barnadas C. Boni V. Latour C. De Monbrison F. Kaiser K. Picot S. (2007). Features of apoptosis in *Plasmodium falciparum* erythrocytic stage through a putative role of PfMCA1 metacaspase-like protein. J. Infect. Dis. 195, 1852–1859 10.1086/518253 17492602

[B61] Meslin B. Beavogui A. H. Fasel N. Picot S. (2011). *Plasmodium falciparum* metacaspase PfMCA-1 triggers a z-VAD-fmk inhibitable protease to promote cell death. PLoS ONE 6:e23867 10.1371/journal.pone.0023867 21858231 PMC3157471

[B62] Mukherjee S. B. Das M. Sudhandiran G. Shaha C. (2002). Increase in cytosolic Ca2+ levels through the activation of non-selective cation channels induced by oxidative stress causes mitochondrial depolarization leading to apoptosis-like death in *Leishmania donovani* promastigotes. J. Biol. Chem. 277, 24717–24727 10.1074/jbc.M201961200 11983701

[B63] Nakagawa A. Shi Y. Kage-Nakadai E. Mitani S. Xue D. (2010). Caspase-dependent conversion of Dicer ribonuclease into a death-promoting deoxyribonuclease. Science 328, 327–334 10.1126/science.1182374 20223951 PMC4313557

[B64] Obeid M. Tesniere A. Ghiringhelli F. Fimia G. M. Apetoh L. Perfettini J. L. Castedo M. Mignot G. Panaretakis T. Casares N. Metivier D. Larochette N. van Endert P. Ciccosanti F. Piacentini M. Zitvogel L. Kroemer G. (2007). Calreticulin exposure dictates the immunogenicity of cancer cell death. Nat. Med. 13, 54–61 10.1038/nm1523 17187072

[B65] Ogden C. A. deCathelineau A. Hoffmann P. R. Bratton D. Ghebrehiwet B. Fadok V. A. Henson P. M. (2001). C1q and mannose binding lectin engagement of cell surface calreticulin and CD91 initiates macropinocytosis and uptake of apoptotic cells. J. Exp. Med. 194, 781–795 10.1084/jem.194.6.781 11560994 PMC2195958

[B66] Paris C. Loiseau P. M. Bories C. Breard J. (2004). Miltefosine induces apoptosis-like death in *Leishmania donovani* promastigotes. Antimicrob. Agents Chemother. 48, 852–859 14982775 10.1128/AAC.48.3.852-859.2004 PMC353131

[B67] Parrish J. Li L. Klotz K. Ledwich D. Wang X. Xue D. (2001). Mitochondrial endonuclease G is important for apoptosis in *C. elegans*. Nature 412, 90–94 10.1038/35083608 11452313

[B68] Parrish J. Z. Xue D. (2003). Functional genomic analysis of apoptotic DNA degradation in *C. elegans*. Mol. Cell 11, 987–996 10.1016/S1097-2765(03)00095-9 12718884

[B69] Peng B. W. Lin J. Lin J. Y. Jiang M. S. Zhang T. (2003). Exogenous nitric oxide induces apoptosis in *Toxoplasma gondii tachyzoites* via a calcium signal transduction pathway. Parasitology 126, 541–550 12866791

[B70] Peters N. C. Egen J. G. Secundino N. Debrabant A. Kimblin N. Kamhawi S. Lawyer P. Fay M. P. Germain R. N. Sacks D. (2008). *In vivo* imaging reveals an essential role for neutrophils in leishmaniasis transmitted by sand flies. Science 321, 970–974 10.1126/science.1159194 18703742 PMC2606057

[B71] Pradelli L. A. Beneteau M. Ricci J. E. (2010). Mitochondrial control of caspase-dependent and -independent cell death. Cell. Mol. Life Sci. 67, 1589–1597 10.1007/s00018-010-0285-y 20151314 PMC11115767

[B72] Rico E. Alzate J. F. Arias A. A. Moreno D. Clos J. Gago F. Moreno I. Dominguez M. Jimenez-Ruiz A. (2009). *Leishmania infantum* expresses a mitochondrial nuclease homologous to EndoG that migrates to the nucleus in response to an apoptotic stimulus. Mol. Biochem. Parasitol. 163, 28–38 10.1016/j.molbiopara.2008.09.007 18940204

[B73] Ronet C. Beverley S. M. Fasel N. (2011). Muco-cutaneous leishmaniasis in the New World: the ultimate subversion. Virulence 2, 547–552 10.4161/viru.2.6.17839 21971185 PMC3260548

[B74] Schmitt M. J. Reiter J. (2008). Viral induced yeast apoptosis. Biochim. Biophys. Acta 1783, 1413–1417 10.1016/j.bbamcr.2008.01.017 18291112

[B75] Sen N. Das B. B. Ganguly A. Mukherjee T. Bandyopadhyay S. Majumder H. K. (2004). Camptothecin-induced imbalance in intracellular cation homeostasis regulates programmed cell death in unicellular hemoflagellate *Leishmania donovani*. J. Biol. Chem. 279, 52366–52375 10.1074/jbc.M406705200 15355995

[B76] Sereno D. Holzmuller P. Mangot I. Cuny G. Ouaissi A. Lemesre J. L. (2001). Antimonial-mediated DNA fragmentation in *Leishmania infantum* amastigotes. Antimicrob. Agents Chemother. 45, 2064–2069 10.1128/AAC.45.7.2064-2069.2001 11408224 PMC90601

[B77] Shi H. Tschudi C. Ullu E. (2006). An unusual Dicer-like1 protein fuels the RNA interference pathway in *Trypanosoma brucei*. RNA 12, 2063–2072 10.1261/rna.246906 17053086 PMC1664728

[B78] Silva R. D. Sotoca R. Johansson B. Ludovico P. Sansonetty F. Silva M. T. Peinado J. M. Corte-Real M. (2005). Hyperosmotic stress induces metacaspase- and mitochondria-dependent apoptosis in *Saccharomyces cerevisiae*. Mol. Microbiol. 58, 824–834 10.1111/j.1365-2958.2005.04868.x 16238630

[B79] Singh G. Jayanarayan K. G. Dey C. S. (2005). Novobiocin induces apoptosis-like cell death in topoisomerase II over-expressing arsenite resistant *Leishmania donovani*. Mol. Biochem. Parasitol. 141, 57–69 10.1016/j.molbiopara.2005.01.014 15811527

[B80] Smirlis D. Duszenko M. Ruiz A. J. Scoulica E. Bastien P. Fasel N. Soteriadou K. (2010). Targeting essential pathways in trypanosomatids gives insights into protozoan mechanisms of cell death. Parasit. Vectors 3, 107 10.1186/1756-3305-3-107 21083891 PMC3136144

[B81] Tang X. Halleck M. S. Schlegel R. A. Williamson P. (1996). A subfamily of P-type ATPases with aminophospholipid transporting activity. Science 272, 1495–1497 10.1126/science.272.5267.1495 8633245

[B82] Uren A. G. O'Rourke K. Aravind L. A. Pisabarro M. T. Seshagiri S. Koonin E. V. Dixit V. M. (2000). Identification of paracaspases and metacaspases: two ancient families of caspase-like proteins, one of which plays a key role in MALT lymphoma. Mol. Cell 6, 961–967 10.1016/S1097-2765(05)00086-9 11090634

[B83] van Zandbergen G. Bollinger A. Wenzel A. Kamhawi S. Voll R. Klinger M. Muller A. Holscher C. Herrmann M. Sacks D. Solbach W. Laskay T. (2006). *Leishmania* disease development depends on the presence of apoptotic promastigotes in the virulent inoculum. Proc. Natl. Acad. Sci. U.S.A. 103, 13837–13842 10.1073/pnas.0600843103 16945916 PMC1564231

[B84] van Zandbergen G. Klinger M. Mueller A. Dannenberg S. Gebert A. Solbach W. Laskay T. (2004). Cutting edge: neutrophil granulocyte serves as a vector for *Leishmania* entry into macrophages. J. Immunol. 173, 6521–6525 15557140 10.4049/jimmunol.173.11.6521

[B85] van Zandbergen G. Luder C. G. Heussler V. Duszenko M. (2010). Programmed cell death in unicellular parasites: a prerequisite for sustained infection? Trends Parasitol. 26, 477–483 10.1016/j.pt.2010.06.008 20591738

[B86] van Zandbergen G. Solbach W. Laskay T. (2007). Apoptosis driven infection. Autoimmunity 40, 349–352 10.1080/08916930701356960 17516227

[B87] Vandivier R. W. Ogden C. A. Fadok V. A. Hoffmann P. R. Brown K. K. Botto M. Walport M. J. Fisher J. H. Henson P. M. Greene K. E. (2002). Role of surfactant proteins A, D, and C1q in the clearance of apoptotic cells *in vivo* and *in vitro*: calreticulin and CD91 as a common collectin receptor complex. J. Immunol. 169, 3978–3986 12244199 10.4049/jimmunol.169.7.3978

[B88] Vassella E. Reuner B. Yutzy B. Boshart M. (1997). Differentiation of African trypanosomes is controlled by a density sensing mechanism which signals cell cycle arrest via the cAMP pathway. J. Cell Sci. 110(Pt 21), 2661–2671 9427384 10.1242/jcs.110.21.2661

[B89] Villalba J. D. Gomez C. Medel O. Sanchez V. Carrero J. C. Shibayama M. Ishiwara D. G. (2007). Programmed cell death in *Entamoeba histolytica* induced by the aminoglycoside G418. Microbiology 153, 3852–3863 10.1099/mic.0.2007/008599-0 17975094

[B90] Wanderley J. L. Moreira M. E. Benjamin A. Bonomo A. C. Barcinski M. A. (2006). Mimicry of apoptotic cells by exposing phosphatidylserine participates in the establishment of amastigotes of *Leishmania (L) amazonensis* in mammalian hosts. J. Immunol. 176, 1834–1839 16424214 10.4049/jimmunol.176.3.1834

[B91] Wang X. Yang C. Chai J. Shi Y. Xue D. (2002). Mechanisms of AIF-mediated apoptotic DNA degradation in *Caenorhabditis elegans*. Science 298, 1587–1592 10.1126/science.1076194 12446902

[B92] Welburn S. C. Maudlin I. (1997). Control of *Trypanosoma brucei brucei* infections in tsetse, *Glossina morsitans*. Med. Vet. Entomol. 11, 286–289 9330261 10.1111/j.1365-2915.1997.tb00408.x

[B93] Zalila H. Gonzalez I. J. El-Fadili A. K. Delgado M. B. Desponds C. Schaff C. Fasel N. (2011). Processing of metacaspase into a cytoplasmic catalytic domain mediating cell death in *Leishmania major*. Mol. Microbiol. 79, 222–239 10.1111/j.1365-2958.2010.07443.x 21166905 PMC3047009

[B94] Zangger H. Mottram J. C. Fasel N. (2002). Cell death in *Leishmania* induced by stress and differentiation: programmed cell death or necrosis? Cell Death Differ. 9, 1126–1139 10.1038/sj.cdd.4401071 12232801

